# Mitochondrial heteroplasmy profiling in single human oocytes by next-generation sequencing

**DOI:** 10.1080/23802359.2017.1365634

**Published:** 2017-08-17

**Authors:** Massimo Ancora, Massimiliano Orsini, Alessia Colosimo, Valentina Russo, Maurilia Marcacci, Maria De Santo, Marco D’Aurora, Liborio Stuppia, Valentina Gatta, Barbara Barboni, Cesare Cammà, Mauro Mattioli

**Affiliations:** a Istituto Zooprofilattico Sperimentale dell’Abruzzo e del Molise “G. Caporale”, Teramo, Italy;; b Facoltà di Bioscienze e tecnologie agro-alimentari e ambientali, Università degli Studi di Teramo, Teramo, Italy;; c Casa di Cura Privata Synergo Pierangeli, Spatocco, Chieti, Italy;; d Dipartimento di Scienze Psicologiche, della Salute e del Territorio, Università “G. D’Annunzio” Chieti-Pescara, Chieti, Italy

**Keywords:** Heteroplasmic mutations, human oocyte, mitochondrial DNA, next-generation sequencing

## Abstract

Mitochondrial DNA (mtDNA) plays a key role in the development of a competent oocyte. Mutations of the mitochondrial genome lead to an altered energetic metabolism with negative effects on oocyte developmental competence. In this study, mtDNA heteroplasmy at an intra-oocyte level and between the different analyzed human oocytes (*n* = 12) was identified by a next-generation sequencing (NGS) protocol previously developed by this research group and submitted to GenBank. This method highlighted, in particular, variants in the genes involved in the respiratory chain providing a direct indication of the cell-specific damage within the mitochondrial genome as predictor of the oocyte quality.

Defects of the mitochondrial DNA (mtDNA) cause an altered energetic metabolism with negative effects on oocyte competence (Gianaroli et al. 2014). Indeed, mitochondrial dysfunction leads to ATP depletion and to oxidative stress with consequent alterations of the oocyte function (Lord and Aitken 2013).

A previously developed next-generation sequencing (NGS) protocol allowed to sequence the entire mitochondrial genome of a single oocyte (Ancora et al. 2017). This novel approach, in the present research, was used to carry out a mitochondrial heteroplasmy profiling in single human oocytes.

For this study, the oocytes (*n* = 12) were collected, after providing written informed consents, from three healthy women aged <35 years with normal BMI undergoing *in vitro* fertilization (IVF) for tubal disease. The entire mtDNA was amplified by using the REPLI-g Mitochondrial DNA Kit (Qiagen, Hilden, Germany). The mtDNA genome sequencing of each oocyte was performed in single end using an Ion Torrent^™^ PGM platform (Thermo Fisher Scientific, Waltham, MA). The mtDNA was depleted during analysis. Raw reads were uploaded on the Orione platform (Cuccuru et al. 2014) for quality trimming, assembling, annotation, and variant analysis. The mtDNA massive sequencing of the 12 oocytes showed a coverage that in average was greater of 1500 ×.

Variant calling returned the identification of heteroplasmic mtDNA variants both at an intra-oocyte level and between the different analyzed oocytes, highlighting, in particular, mutations in the genes involved in the respiratory chain (ND2, ND4, ND5, COX2, ATP8, and CYTB). To the best of our knowledge, this is the first study that has investigated the whole mtDNA and heteroplasmy in individual human oocytes using NGS. It is important to realize the full potential of high-throughput sequencing that could be confidently applied for the advancement of mitochondrial research related to oocyte quality.

The mitochondrial DNA sequences were submitted to GenBank under the accession numbers KX061446, KX061447, KX061448, KX061449, KX061450, KX061451, KX061452, KX061453, KX061454, KX061455, KX061456, and KX061457.
